# Genetic structure and origin of emu populations in Japanese farms inferred from large-scale SNP genotyping based on double-digest RAD-seq

**DOI:** 10.1038/s41598-024-57032-y

**Published:** 2024-03-24

**Authors:** Yuichi Koshiishi, Kenta Wada

**Affiliations:** 1 NODAI Genome Research Center, Tokyo University of Agriculture, Setagaya, Tokyo 156-8502 Japan; 2 Faculty of Bioindustry, Tokyo University of Agriculture, Abashiri, Hokkaido 099-2493 Japan

**Keywords:** Agricultural genetics, Animal breeding

## Abstract

The emu is a novel poultry species in Japan. However, Japanese farmed emu populations have reduced genetic diversity owing to inbreeding. We have previously suggested that there are genetic resources in the Tohoku Safari Park (TSP) and Fuji/Kakegawa Kachoen Garden Park (FGP/KGP) to extend the genetic diversity of commercial emu farms based on microsatellite (SSR) and mitochondrial DNA. However, those markers provide relatively poor information. Thus, we investigated the genetic structure of farmed Japanese populations based on a large-scale genotyping system using RAD-seq and verified the usefulness of TSP and FGP/KGP as genetic resources for expanding genetic diversity. Admixture, phylogenetic, and principal component analyses based on 28,676 SNPs showed that TSP individuals were ancestors in the Okhotsk Emu Farm (OEF). FGP/KGP individuals showed a unique genetic component that differed from that of the others. We have previously reported that the mitochondrial haplotypes of FGP/KGP were shared with an isolated wild population in eastern Australia. These results suggest that FGP/KGP individuals originated from an eastern Australia isolated population different from other populations including ancestral of OEF/TSP. Our results would provide information for the development of Japanese emu farms and industry and for the conservation of genetic resources in the Australian wild emu.

## Introduction

The emu (*Dromaius novaehollandiae*) is a ratite species originally found in Australia. For a few decades, the emu has been expected to become a novel poultry breed that produces low-fat meat^1^, hypoallergenic eggs^2,3^, and oil containing a high proportion of unsaturated fatty acids^4,5^. In particular, emu oil purified from subcutaneous fat has anti-inflammatory properties^6–8^ and thus has been utilized as a material for skincare products with high economic value^9^. The industrial use of emus was began in Australia in the 1970s, followed by USA, China, India, Canada and Japan^4,9–11^.

While products from emus are highly useful, their production traits have not genetically improved owing to the short history of their domestication, indicating that it might be the cause of low effective production in industries. Thus, the economic traits of emus should be genetically improved to further develop the emu industry. Although selective breeding based on marker-assisted selection is an effective strategy for rapid genetic improvement in livestock, maintaining high genetic diversity in populations may be required.

In Japan, there are few small populations used for farming and ornamental purposes (Fig. S1). The Okhotsk Emu Farm (OEF; Abashiri, Hokkaido, Japan) is the largest farm in Japan, with more than 500 individuals, which was established approximately 20 years ago, with emus introduced from farms in the USA, Australia, and Japan^12^. Tohoku Safari Park (TSP; Nihonmatu, Fukushima, Japan) has only 19 individuals of unknown origin with the sole purpose of exhibition^12,13^. Fuji Kachoen Garden Park (FGP; Fujinomiya, Shizuoka, Japan) and Kakegawa Kachoen Garden Park (KGP; Kakegawa, Shizuoka, Japan) breed 30 and 14 individuals, respectively, and were predicted to have imported from African or American farms according to the breeder’s comments.

Based on mitochondrial DNA (mtDNA) and microsatellite DNA (SSR) polymorphisms, we have previously reported that genetic diversity within populations tends to be reduced through inbreeding^5,12,13^. We also found that some farm populations in Japan harbored different genetic components. Our previous study suggested that TSP, FGP, and KGP could be distinct populations from OEF and other two farms^5^. These results indicate that TSP, FGP, and KGP, which are composed of a small number of individuals for ornamental use, could become useful genetic resources for extending the genetic diversity of Japanese production farms. However, mtDNA analysis is limited to maternal information and SSR markers are not always widely distributed. In particular, it is necessary to use numerous SSR markers to robustly understand the genetic relationships among populations. Therefore, the genetic relationships among farmed Japanese emu populations remain unclear.

Restriction site-associated DNA sequencing (RAD-seq) is a robust and effective method for obtaining genome-wide SNP genotypes in a large number of samples and is used for population genetics^14,15^, quantitative trait locus (QTL) analysis^16,17^, and marker development^18,19^ in eukaryotes, including non-model organisms. In this study, we performed large-scale SNP genotyping using RAD-seq and compared the genetic structures of three Japanese emu populations which were observed marked genetic differentiation in our previous report. Here, we revealed the detailed genetic structures within populations and relationships among populations and found that KGP and FGP have clearly different genetic components from the others, and they might have originated from an isolated small Australian population. While some TSP individuals may share identical genetic components with OEF, a significant proportion could belong to an independent population. To the best of our knowledge, this is the first report to attempt population genetic study based on RAD-seq in the emu. In addition to genetic relationships among Japanese farmed populations, the present and previous studies indirectly suggest that a portion of Japanese emu populations may have originated from a minor wild population in Australia.

## Materials and methods

### Sample

In a previous study, we suggested that TSP and FGP/KGP are genetically different from OEF based on analyses using SSR markers and mtDNA haplotypes^5^. Therefore, we used samples derived from the same populations as in our previous studies and included a large number of individuals with OEF as controls.

Liver tissues were collected from 95 and 100 birds slaughtered in 2016 (OEF2016) and 2017 (OEF2017) at OEF, respectively. Feather pulp samples (n = 58) were randomly collected from 19, 25, and 14 individuals in the TSP, FGP, and KGP groups, respectively. Genomic DNA (n = 253) was isolated from liver tissues and feather pulp using ISOGENOME (Nippon Gene, Tokyo, Japan) according to the manufacturer’s instructions.

All procedures involving animals met the guidelines described in “The Proper Conduct of Animal Experiments,” proposed by the Science Council of Japan and approved by the Ethical Care and Use of Animals Committee at the Tokyo University of Agriculture (approval number: 270049, 280,002, 290,096, 300,126, 2,019,109, 2,022,132, and 2,023,156). The study was carried out in compliance with the ARRIVE guidelines.

### RAD-seq library construction and Illumina sequencing

A sequence library was constructed using the flexible double digest RAD-seq (flexible ddRAD-seq) method^20^. Extracted DNAs (10–100 ng) were digested using two kinds of restriction enzymes, *Eco* RI (5′ GAATTC3′) and *Hind* III (5′ AAGCTT3′), for 2 h at 37 °C. Digested DNA fragments ligated to custom adaptors that have cleavage site of *Hind* III anneals P5 adaptor (5′- AGC TCT GTC TCT TAT ACA CAT CTG ACG CTG CCG ACG A-3′ [underline indicated annealing site]), and the site of *Eco*RI anneals P7 adaptor (5′- AAT TCT GTC TCT TAT ACA CAT CTA ATC A-3′ [underline indicated annealing site]) by T4 DNA ligase (Takara Bio, Otsu, Japan). The adaptor-ligated DNA fragments were amplified via PCR using PrimeSTAR HS (Takara Bio, Shiga, Japan) with a unique dual-index primer. Following library construction, DNA library sequencing was performed on a NextSeq 1000 (Illumina, San Diego, CA, USA) using 150-bp paired-end reads with custom primers. FASTQ file was formatted using bcl2fastq. Our data were submitted to the DDBJ Sequence Read Archive (DRA) under accession no. DRR519049-DRR519301 (Table S1). FastQC ver. 0.11.9 (http://www.bioinformatics.babraham.ac.uk/projects/fastqc/) and Trimmomatic ver. 0.39^21^ were used to perform quality check (QC), quality trimming, and read pairing.

Trimming parameters were following command: “ILLUMINACLIP TruSeq3-PE-2,” 2:30:10:2; “LEADING,” 20; “TRAILING,” 20; “CROP,” 148; “HEADCROP,” 13; “SLIDINGWINDOW,” 4:15; and “MINLEN,” 50. All filtered and paired reads were mapped to the reference emu genome (GenBank GCA_016128335.1) using the Burrows-Wheeler Aligner (BWA) software^22^. The mapped data output as SAM-format files was sorted and converted to BAM format using SAMtools ver. 1.14^23^.

### SNP detection and evaluation of genetic diversity

SNPs were detected using the ref-map.pl script in Stacks ver. 2.61^24^ with − r 0.5 and − p 5 options from each mapping data set. The number of polymorphic loci, number of private alleles, expected heterozygosity (*H*_E_), observed heterozygosity (*H*_O_), and *F*_IS_ values were calculated using populations software in the Stacks script. Parentage analysis for the detection of sibships and the inbreeding effect was visualized using Colony2 software^25^ (https://www.zsl.org/about-zsl/resources/software/colony).

### Construction of phylogenetic tree

The genetic distance (*F*_ST_) among farms was calculated using Populations 1.2.32 software (https://bioinformatics.org/~tryphon/populations/) with a genepop-format file outputted by the populations software in Stacks. The genetic distances were computed among all individuals and phylogenetic trees were constructed using BEAST2 ver. 1.7.4. The DensiTree package in the R ver. 4.2.2 (https://www.r-project.org/) was used to visualize multi-phylogenetic trees.

### Admixture analysis

The most likely number of ancestors were predicted based on the PLINK format file using ADMIXTURE ver. 1.3.0^26^ (https://dalexander.github.io/admixture/index.html) under default conditions. The bar plot was exported using the Pophelper and ggplot2 packages in the R software.

### Principal coordinate analysis (PCoA) with tSNE plot

The genotype data formatted by the VCF file were manipulated using the vcfR package in R software. After PCoA was performed for up to 50 dimensions based on the loaded SNP information, dimensionality reduction was performed to calculate the t-distributed stochastic neighbor embedding (tSNE). The tSNE plot was drawn using the ggplot2 package in R software.

## Results

### SNP detection and genetic diversity

In five populations, 3,166,876 loci were commonly mapped to the reference sequence, with 28,676 SNPs detected at a rate of over 50% of samples in each population. Among these, 5198, 22,271, 25,484, 13,469, and 6825 polymorphic sites were detected in TSP, OEF2016, OEF2017, FGP, and KGP, respectively (Table 1). The polymorphic rates of the 3,166,876 mapped reads were 0.16%, 0.70%, 0.80%, 0.43%, and 0.22% for TSP, OEF2016, OEF2017, FGP, and KGP, respectively. The number of private alleles were 69, 1260, 3259, 636, and 154 in TSP, OEF2016, OEF2017, FGP, and KGP, respectively. Therefore, OEF possessed higher genetic variation than the other populations, in contrast to the lower values in the TSP and KGP populations. The expected and observed heterozygosities (*H*_E_/*H*_O_) of TSP, OEF2016, OEF2017, FGP, and KGP showed low values, which were 0.025/0.018, 0.035/0.028, 0.040/0.034, 0.039/0.029, and 0.033/0.024, respectively. The inbreeding coefficients (*F*_IS_) of the TSP, OEF2016, OEF2017, FGP, and KGP were 0.320, 0.198, 0.175, 0.237, and 0.305, respectively. These results suggest that all tested Japanese farmed populations tend to inbreed and that TSP is in the most profound condition, consistent with our previous reports^5,27^. Pedigree analysis using Colony2 software showed that there were three full-sib pairs and 69 half-sibs in the analyzed 253 individuals (Fig. S2). Among them, 3 full-sibs and 53 half-sibs were found in the TSP, indicating that the inbreeding of this population progressed.

**Table 1:** General genetic parameters in study populations based on SNPs detected via RAD-seq.

Pop ID	SNP sites	% of polymorphic loci	Private alleles	% of private alleles	Pi	*H*_O_	*H*_E_	*F*_IS_	*N*_*e*_
TSP	5198	0.16	69	1.327	0.026	0.018	0.025	0.320	18.9
OEF2016	22,271	0.70	1260	5.658	0.035	0.028	0.035	0.198	94.1
OEF2017	25,484	0.80	3259	12.788	0.041	0.034	0.040	0.175	99.8
FGP	13,469	0.43	636	4.722	0.039	0.029	0.039	0.273	24.6
KGP	6825	0.22	154	2.256	0.034	0.024	0.033	0.305	11.4

TSP, OEF2016/2017, FGP, and KGP represent Tohoku Safari Park, Okhotsk, Fuji Kachoen Garden Park, and Kakegawa Kachoen Garden Park, respectively. Pi, *H*_O_, *H*_E_, *F*_IS_ and *N*_e_ represent allele frequency, observed heterozygosity, expected heterozygosity, inbreed coefficient, and effective population size, respectively.

### Phylogenetic trees

**Figure 1:**
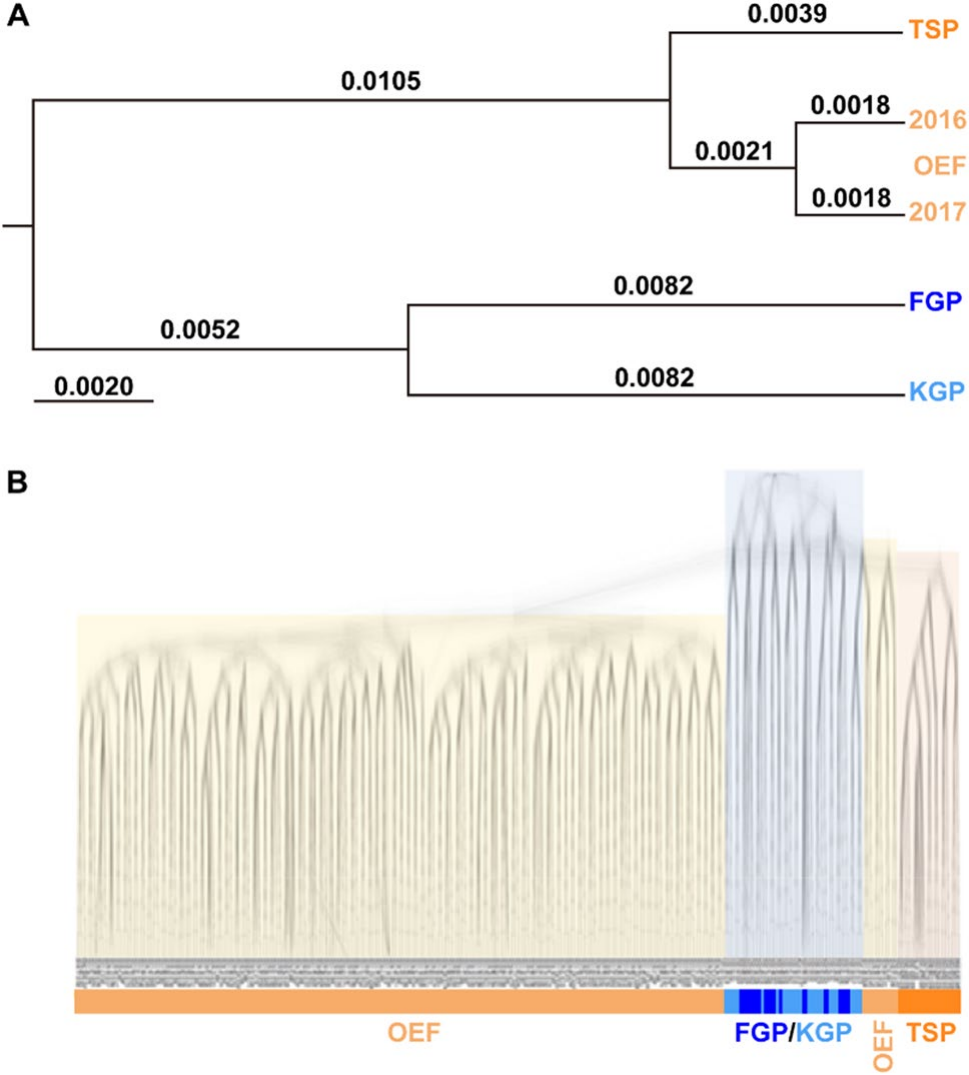
Phylogenetic trees of Japanese farmed emu populations based on SNP genotypes. (**A**) Phylogenetic tree among four populations based on *F*_ST_ calculated using SNP genotypes. *F*_ST_ values were indicated in each branch. (**B**) Phylogenetic trees of 253 emu individuals based on the Beast software calculated using SNP genotypes. The yellow, blue, and orange node (**A**, **B**) and blanch (**B**) colors indicated OEF, KGP/FGP, and TSP, respectively. OEF, Okhotsk Emu Farm; TSP, Tohoku Safari Park; FGP, Fuji Kachoen Garden Park; KGP, Kakegawa Kachoen Garden Park.

Genetic distance (*F*_ST_) among the populations ranged from 0.004 to 0.037 (Table S2). The lowest and highest *F*_ST_ values were observed between OEF2016 and OEF2017 (0.004) and between KGP and TSP (0.037), respectively. The phylogenetic tree based on* F*_ST_ showed that FGP and KGP formed a clade with a relatively high genetic distance between them, which was distinct from the clade composed of OEF2016, OEF2017, and TSP (Fig. 1A). The phylogenetic tree, based on the genetic distance among 253 individuals derived from all tested populations, showed four clusters constructed by TSP, FGP/KGP, and separated OEFs (Fig. 1B). In the cladogram shown in Fig. 1B, the lines were more clearly drawn for reliable divergences than for uncertain divergences. FGP and KGP were predicted to be separate from the common ancestors of all the tested Japanese emu farm populations in the early stages. A few individuals derived from the OEF clustered into a clade different from many other members and had a closed relationship with the TSP population. These results suggest that the origin of FGP/KGP may be from a different Australian population than the others in Japan, and OEF is composed of two divergent groups, one of which shares common ancestors with a few individuals in the TSP.

### Admixture analysis

**Figure 2:**
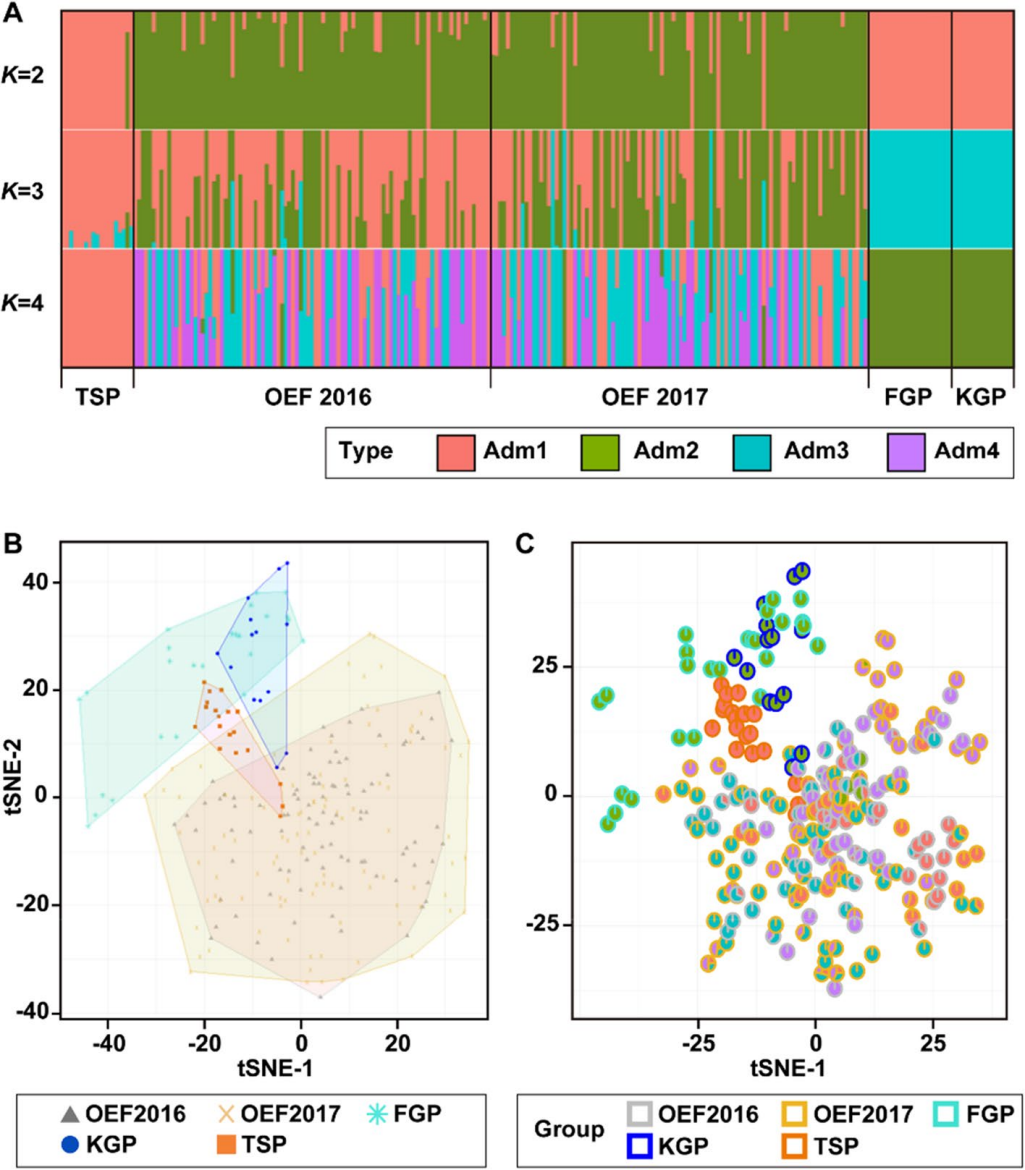
Genetic structure and relationships among Japanese emu farmed populations. (**A**) ADMIXTURE analysis of four emu populations based on SNP genotype data. Each genotyped emu is indicated by a single vertical line divided into *K* colors (red, green, ice blue, and purple), where *K* is the putative number of ancestors. (**B**) tSNE plot of 253 emu individuals based on SNP data. The five groups were bundled by different colored polygons. Gray (triangles), yellow (cross marks), ice blue (snowflakes), blue (circles), and orange (squares) represent OEF2016, OEF2017, FGP, KGP, and TSP, respectively. (**C**) tSNE pie plot of 253 emu individuals based on SNP genotype data. Each individual points showed pie chart of divided genetic cluster via Admixture analysis (**A**). Colored outer boundary of pie charts by gray, yellow, ice blue, blue, and orange indicated individuals derived from OEF2016, OEF2017, FGP, KGP, and TSP, respectively. OEF, Okhotsk Emu Farm; TSP, Tohoku Safari Park; FGP, Fuji Kachoen Garden Park; KGP, Kakegawa Kachoen Garden Park.

To estimate genetic variability within and divergence among populations, structural analysis was performed using ADMIXTURE software. At *K* = 2, the genetic structure showed that TSP and FGP/KGP had similar components, which were distinct from those of OEF (Fig. 2A, *upper*). In *K* = 3, although TSP and FGP/KGP were predominantly represented in monochrome, respectively, as they were *K* = 2, they exhibited distinct genetic components between them (Fig. 2A, *middle*). OEF has a complex genetic structure, including the TSP and a few FGP/KGP components. At *K* = 4, TSP was completely represented by a single color (Adm1), and OEF had all the genetic components derived from TSP and FGP/KGP (Adm2), in addition to their own unique components (Fig. 2A, *bottom*). These results indicate that TSP and FGP/KGP have largely different genetic components and that OEF was constructed by mixing ancestral the sharing with TSP and a few FGPs/KGPs.

### PCoA with tSNE plot

The t-distributed stochastic neighbor embedding (tSNE) plot based on SNP genotypes grouped the OEF, TSP, and FGP/KGP populations, except for a few individuals (Fig. 2B). Similar to the results shown in Fig. 1, OEF was widely distributed on these reduced dimensions, indicating that it had various genetic variations compared with the other populations. Almost all the KGP individuals overlapped with the FGP group. The distribution of tSNE in the OEF and FGP groups was completely separated, and they were widely distant. In contrast, the distribution of a few individuals from the TSP and KGP groups overlapped with that of the individuals from the OEF group. A large proportion of TSP individuals were collectively distributed adjacent to the FGP/KGP group.

Next, we integrated the results from the admixture analysis and the tSNE plot, as shown in Fig. 2C. The pie charts of each individual assigned by the admixture analysis were mapped onto the tSNE plot to visualize the genetic structure and genetic distance among all samples (Fig. 2C). Consistent with the above results, the FGP (ice blue colored frame) and KGP (blue colored frame) groups formed distinct clusters from the OEF group (gray- and yellow-colored frames), and their genetic components were composed of only Adm2 (green colored pie chart), which was not observed in the other populations. The OEFs were distributed into three clusters (Adm1, Adm3, and Adm4) when the putative number of ancestors (*K*) was four in the admixture analysis (Fig. 2A). The scatter tSNE plot-integrated clustering information obtained from the admixture analysis showed that OEF was widely spread from coordinates approximately − 25 in tSNE-1 to 25 in tSNE-2, with multiple clusters for each genetic component. The OEF population comprises three subgroups: Adm1 (represented by an orange pie chart), Adm3 (depicted as an ice blue pie chart), and Adm4 (represented by a purple pie chart). These subgroups were derived from various ancestors and exhibited a significant spread on the tSNE plot, suggesting a greater degree of genetic diversity than other populations.

The Adm1, which was occupied by individuals derived from TSP when *K* = 4, displayed distinct distributions near the KGP/FGP clusters and within the OEF cluster on coordinates (− 20.0, 21.4) and (− 3.7, − 1.7) in the tSNE-1 and tSNE-2 dimension, respectively. Some individuals from the TSP exhibited overlapping plots within the OEF population. Therefore, it can be inferred that some TSP individuals share common ancestral origins with the OEF population.

## Discussion

### Genetic diversity of Japanese farmed populations

In our previous studies, Japanese farmed emu populations were clustered into genetically distinct groups based on SSR marker and mtDNA analyses^5,12,13^. The TSP and FGP/KGP populations exhibited genetic components distinct from the OEF population. Therefore, we previously suggested that TSP and FGP/KGP should be constructed from specific ancestral populations. To verify our previous hypothesis, in the present study, we investigated the genetic structure among those populations with OEF based on a large-scale SNP genotyping by RAD-seq analysis, which has a higher resolution compared to that of SSRs and mtDNA.

The number of polymorphic loci that could be mapped to the reference genome was lower in the TSP and KGP populations than that in the other populations (Table 1). In addition, a smaller number of private alleles was observed in TSP/KGP. It is possible that genetic diversity in these populations is low. Because TSP and KGP were composed of a small number of individuals, these results might be derived from a limited number of founders or caused by the reduction of minor alleles due to inbreeding and/or genetic drift, consistent with our previous studies using SSR markers^5,12^. Alternatively, the small number of private alleles detected in TSP/KGP could be explained by their small population sizes. Therefore, we cannot conclude that genetic diversity in lower in TSP/KGP than in OEF.

### Genetic relationships among Japanese farmed populations

In the phylogenetic tree based on the genetic distance among the four populations, FGP and KGP independently diverged from TSP/OEF (Fig. 1A), which is consistent with previous studies^5,12,13^. Unlike previous studies, the TSP population showed a close relationship with the OEF population^5^. The cladogram constructed using the genotypes from 253 individuals also showed that the clade of the FGP/KGP group was clearly separated from that of the other populations (Fig. 1B). Interestingly, FGP and KGP were separated from the other populations before the divergence between TSP and OEF. This result indicates that some TSP individuals were derived from common ancestors to those of OEF, unlike FGP and KGP. Hence, we propose that the sources of the FGP and KGP populations were different from those of the Australian population from the OEF/TSP. Therefore, genetic differentiation observed in these populations may not be attributable to genetic drift or inbreeding.

The Japanese farmed populations were largely divided into three clusters, represented by the OEF, TSP, and FGP/KGP groups, with *K* = 3 and 4 as the putative number of ancestors (Fig. 2A). In *K* = 4, although TSP and FGP/KGP showed a single cluster, OEF was composed of multiple genetic components. These results suggest that OEF is an admixed population with multiple ancestors and thus maintains a higher genetic diversity than the other three populations composed of a single cluster. Meanwhile, microsatellite analysis in our previous study showed that the OEF population had a “moderate number of alleles,” which was higher than that of TSP, FGP, and KGP, supporting the findings of the present study.

Recently, genome-wide genotyping systems, including RAD-seq, have revealed the genetic relationships among populations and hidden genetic structures in many wild organisms and livestock^29–31^. Using RAD-seq, we successfully identified genetic divergence among populations and discovered rare genetic components in the Japanese emu. Thus, this study further supports the utility of RAD-seq-based for population genetics analyses of farmed animals.

### Putative origins of Japanese farmed populations

A large proportion of individuals derived from FGP and KGP showed overlapping plots in the tSNE analysis (Fig. 2B). The tSNE scatter plot and admixture analysis also indicated that FGP/KGP have common ancestors, which is consistent with comments from breeders in these populations. In the tSNE plot, the KGP group appeared to be more tightly clustered than the FGP group, reflecting a lower rate of private alleles and pi in KGP than those in FGP (Table 1). Conversely, FGP was predicted to maintain a higher genetic diversity than KGP, even though they exhibited similar population sizes, reflecting widespread plotting on the tSNE in FGP individuals. However, it is unknown why FGP maintain higher genetic diversity than KGP despite similar population sizes and identical sources.

Davis et al*.*^28^ revealed that a wild emu population on the NSW North Coast (NNC) was in reproductive isolation from other populations and suggested that this was caused by geographic isolation due to the presence of the Great Dividing Range. The NNC population showed clear differentiation from the others in the STRUCTURE analysis in their study, and the predominant mtDNA haplotype was H8, which has rarely been observed in other populations^28^. To predict the origin of farmed Japanese populations, our previous results were compared with those reported by Davis et al*.* (2022). The Hap-d corresponded to H8^28^ and is the most frequently detected haplotype in FGP/KGP^5^ and is clearly differentiated from other Japanese populations (Figs. 1 and 2). These facts imply that the primary source of FGP/KGP was the NNC, a wild population in eastern Australia. However, because mtDNA is transmitted by maternal inheritance, it is necessary to compare SNP profiles between FGP/KGP and NNC to verify our hypothesis.

Most TSP individuals were aggregately plotted at an intermediate position between the OEF and FGP groups (Fig. 2B,C). To understand the detailed genetic relationship between TSP and other populations, we generated a 3D scatter plot depicting the tSNE1-3 components, which indicated that TSP was grouped independently from other populations (Fig. S3). Notably, Adm1, represented by all individuals from the TSP, was distributed near the KGP/FGP and within the OEF clusters (Fig. 2C). These results suggest that part of the TSP was constructed using genetic components derived from OEF and unknown relative populations. In addition, parentage analysis based on SNP genotypes indicated that almost all TSP individuals were in full-sib or half-sib pairs (Fig. S2). This indicates that the TSP population is advancing to a higher level of inbreeding within close relatives. We have previously suggested that the TSP was constructed using a unique genetic component different from OEF based on mtDNA and SSR analyses. Notably, a large proportion of TSP individuals harbored Hap-c, a very rare mtDNA haplotype that has not been found in previous studies^5,12,13^. However, large-scale SNP genotyping based on RAD-seq revealed that TSP harbored a lower number of private alleles than the others, and some of these individuals were included in a cluster composed of OEF members (Table 1, Fig. 2A). The tSNE plot and parentage analysis showed that individuals with TSP shared the same genotypes at many loci within the population, indicating that they may have experienced a bottleneck in the past (Fig. 2B,C). Therefore, we predicted that the ancestors of the TSP population partially originated from individuals common to OEF.

Taking these results into consideration, we propose two hypotheses regarding the origins of TSP as follows: i) TSP lost minor alleles through the bottleneck effect and inbreeding, resulting in the deflection of allele frequencies, and then Hap-c, a minor haplotype in the Australian population, was extended into TSP by founder effects. ii) The Admixture and PCoA analyses demonstrated that TSP was an independent population from the others, despite sharing some OEF ancestors. Accordingly, the origin of the TSP provisionally designated as *ancTSP* might be an isolated population in the Australian wild emus, as well as in the NNC. This hypothesis is supported by the presence of rare genetic components and an mtDNA haplotype, which have rarely been detected in other populations, including Australia^5,12,13^. Thus, it is possible that TSP partially originated from a rare Australian mainland population after the isolation of NNC, a putative origin of FGP/KGP, from the major populations (Fig. 1B). If this hypothesis is correct, it would explain the independence of TSP, despite harboring a few genetic components derived from OEF. The survival of *ancTSP* is highly desirable for the conservation of genetic resources as a cryptic population that has never been discovered in the emu. In conclusion, further analyses based on large-scale SNP genotyping of Australian wild and farmed populations, including extinct species, are needed.

## Conclusions

In conclusion, the present study revealed the genetic structure within and the relationships among Japanese emu populations based on genome-wide SNP genotyping. The OEF is the largest population in Japan and has the highest genetic diversity among all the tested populations. FGP and KGP were clearly differentiated from OEF, suggesting that they originated from different populations in Australia. TSP may be composed of a common ancestor of OEF and its unique genetic component is caused by the loss of genetic diversity due to inbreeding. Alternatively, the TSP may be independent of the Japanese farmed population derived from rare Australian individuals. To the best of our knowledge, our study is the first to predict the genetic relationships among populations of Japanese farmed emus and their origins. These findings would provide valuable insights not only for effective emu breeding to extend genetic diversity in Japan but also for the conservation of genetic resources in wild and farmed Australian emus.

### Supplementary Information

Supplementary Information.

## Data Availability

Our data were submitted to the DNA Data Bank of Japan (DDBJ) Sequence Read Archive (DRA) under accession no. DRR519049-DRR519301. The datasets are available in following link; https://ddbj.nig.ac.jp/resource/bioproject/PRJDB17187.
