# Supplementary material for: Genetic structure and origin of emu populations in Japanese farms inferred from large-scale SNP genotyping based on double-digest RAD-seq
Source: Sci Rep. 2024 Mar 24;14:6982. doi: 10.1038/s41598-024-57032-y (PMC10961305; doi:10.1038/s41598-024-57032-y)
Supplement: Supplementary file 1 — Supplementary Information. [file 41598_2024_57032_MOESM1_ESM.docx]

**
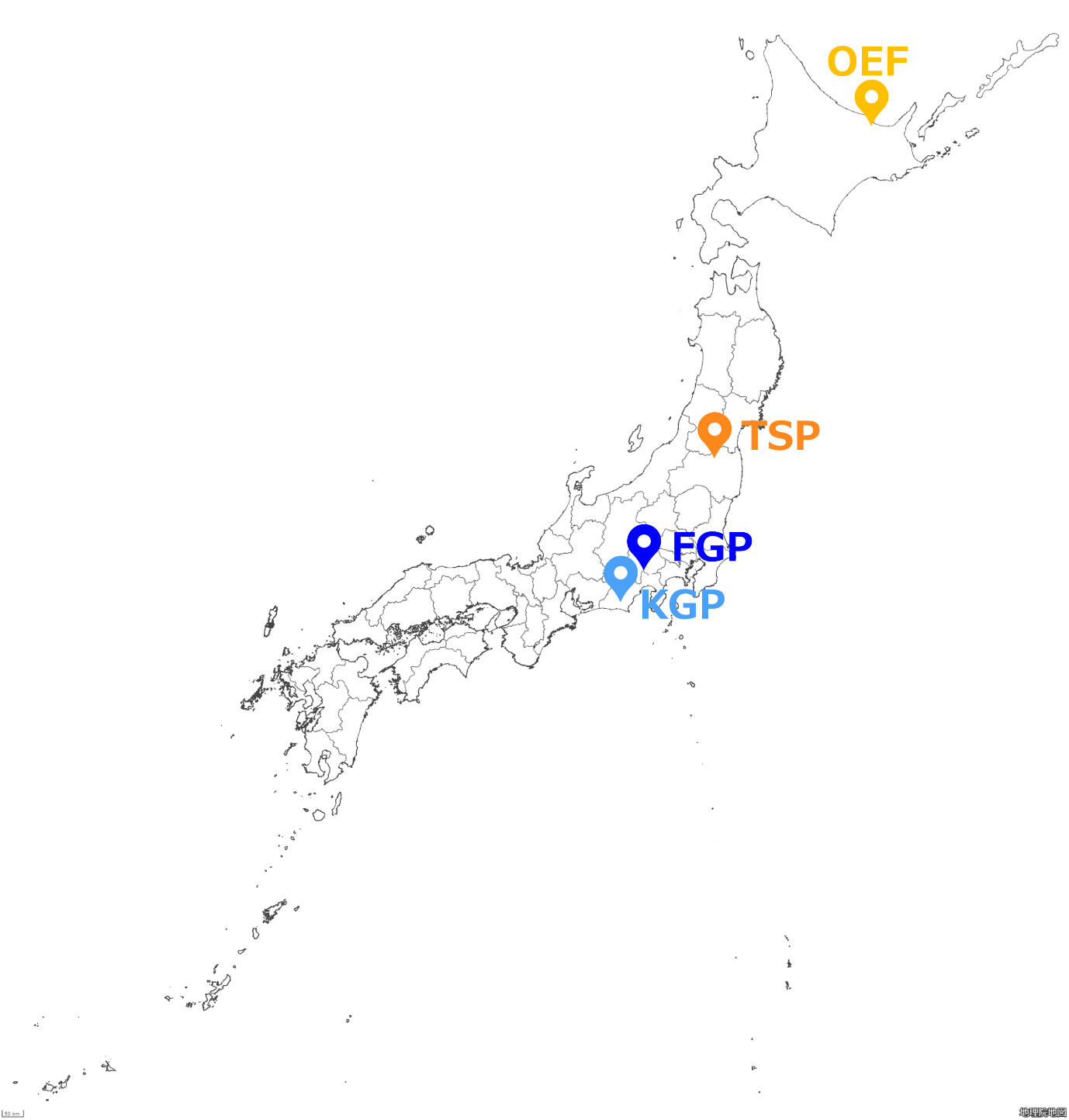
Koshiishi and Wada. Supplementary figure 1.** The places collected feather pulps in this study. The spots colored by yellow, orange, blue and light blue on the base map of Japanese island indicated Okhotsk Emu Farm (OEF), Thohoku Safari Park (TSP), Fuji Garden Park (FGP), and Kakegawa Garden Park (KGP), respectively. The map data was sourced Geospatial Information Authority of Japan (https://www.gsi.go.jp/ENGLISH/index.html).


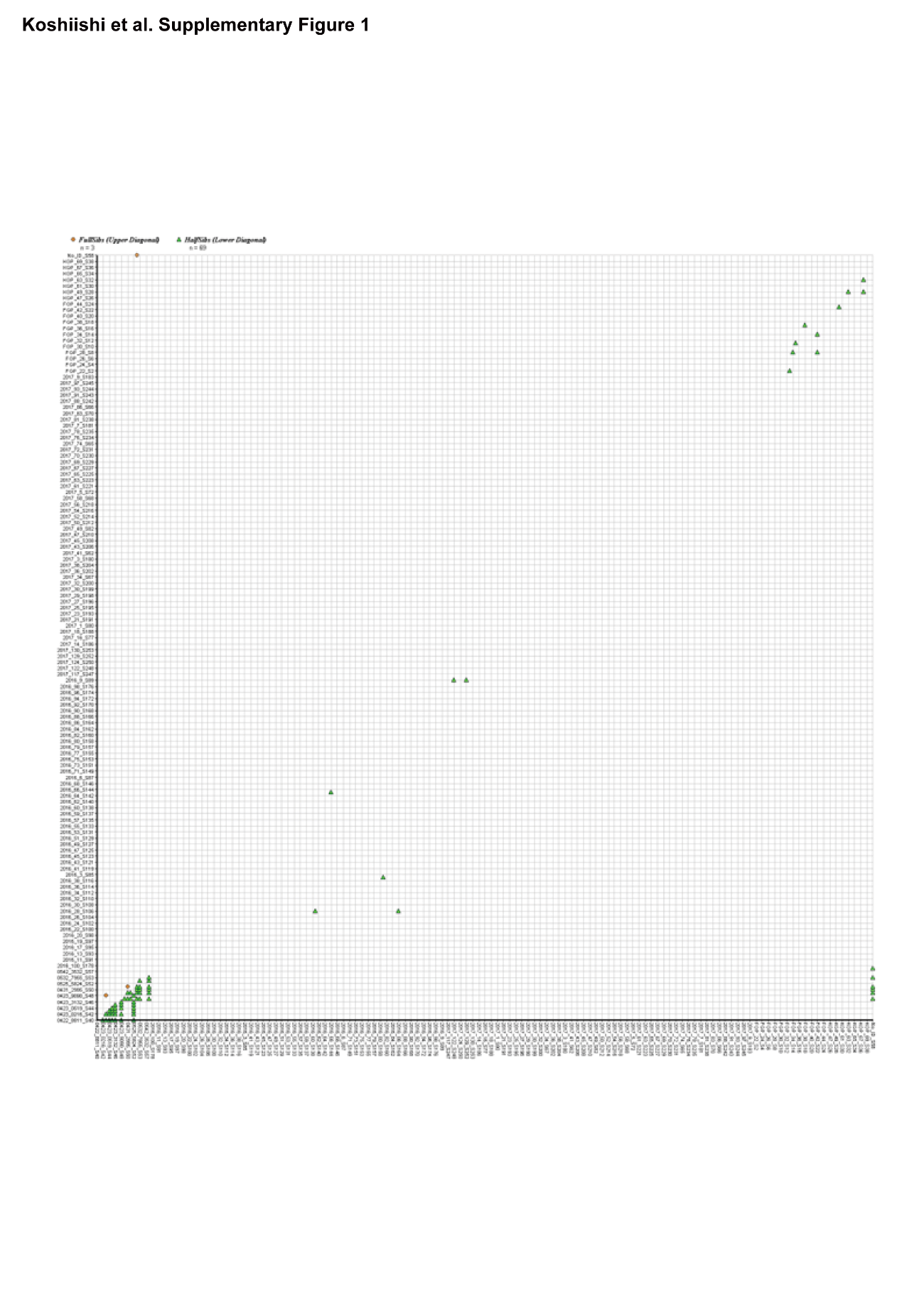
**Koshiishi and Wada. Supplementary figure 2.** Pedigree analysis using Colony2 software showed that there were three full-sib pairs and 69 half-sibs in the analyzed 253 individuals.


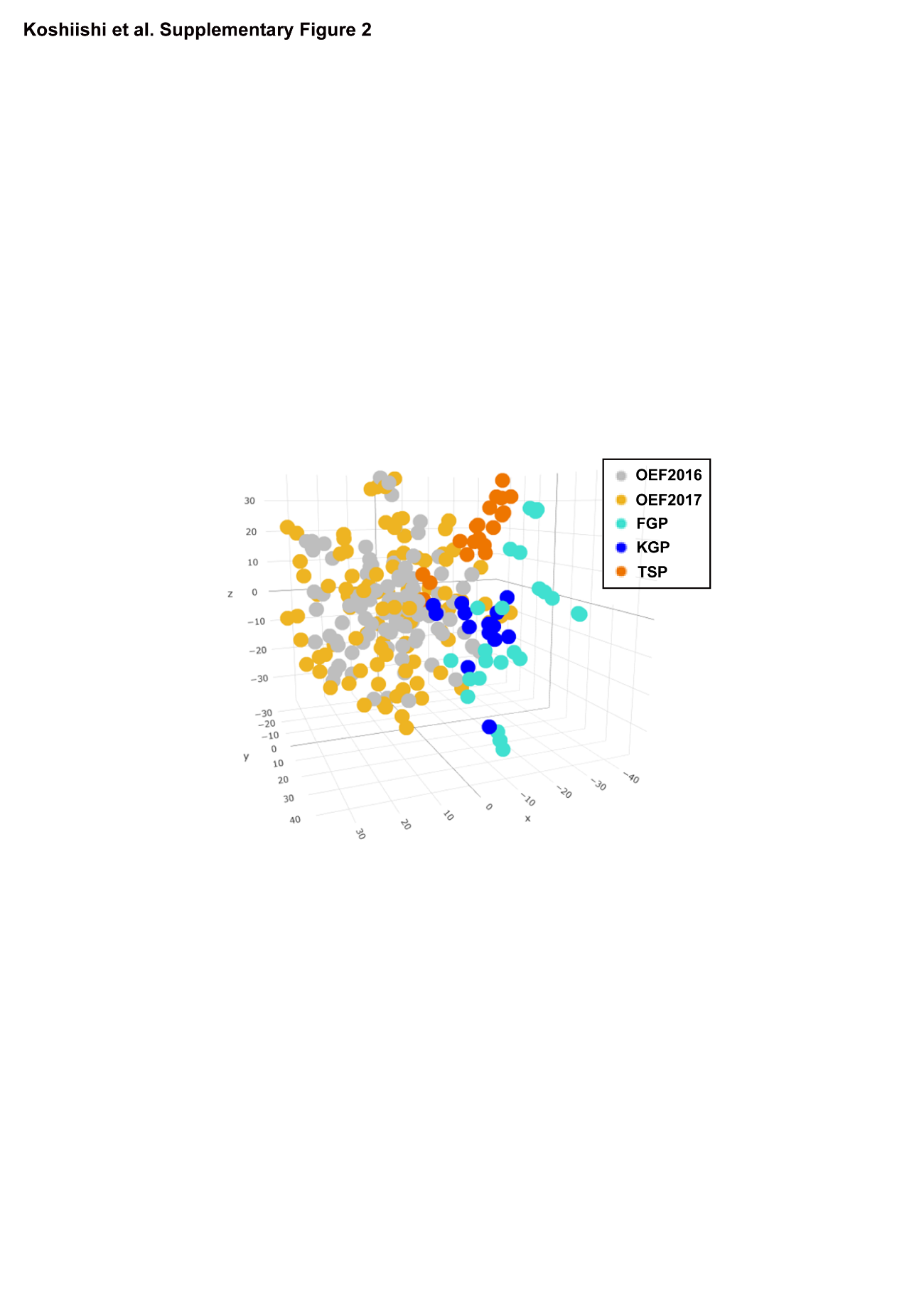


**Koshiishi and Wada. Supplementary figure 3.** The 3D scatter plot depicting the tSNE1-3 components.

| **Supplementary Table 1**. Information of samples used in this study | | | |
| --- | --- | --- | --- |
| **Sample name** | **Population** | **Sampling year** | **DRR** |
| 0422_8811 | TSP | 2015 | DRR519049 |
| 0423_0179 | TSP | 2015 | DRR519050 |
| 0423_0216 | TSP | 2015 | DRR519051 |
| 0423_0450 | TSP | 2015 | DRR519052 |
| 0423_0519 | TSP | 2015 | DRR519053 |
| 0423_2598 | TSP | 2015 | DRR519054 |
| 0423_3132 | TSP | 2015 | DRR519055 |
| 0423_7517 | TSP | 2015 | DRR519056 |
| 0423_9090 | TSP | 2015 | DRR519057 |
| 0430_9973 | TSP | 2015 | DRR519058 |
| 0431_2995 | TSP | 2015 | DRR519059 |
| 0431_6174 | TSP | 2015 | DRR519060 |
| 0525_5824 | TSP | 2015 | DRR519061 |
| 0532_1045 | TSP | 2015 | DRR519062 |
| 0532_7955 | TSP | 2015 | DRR519063 |
| 0533_1635 | TSP | 2015 | DRR519064 |
| 0542_3532 | TSP | 2015 | DRR519065 |
| 0542_5400 | TSP | 2015 | DRR519066 |
| No_ID | TSP | 2015 | DRR519067 |
| 2016_1 | OEF | 2016 | DRR519068 |
| 2016_10 | OEF | 2016 | DRR519069 |
| 2016_100 | OEF | 2016 | DRR519070 |
| 2016_11 | OEF | 2016 | DRR519071 |
| 2016_12 | OEF | 2016 | DRR519072 |
| 2016_13 | OEF | 2016 | DRR519073 |
| 2016_14 | OEF | 2016 | DRR519074 |
| 2016_17 | OEF | 2016 | DRR519075 |
| 2016_18 | OEF | 2016 | DRR519076 |
| 2016_19 | OEF | 2016 | DRR519077 |
| 2016_20 | OEF | 2016 | DRR519078 |
| 2016_21 | OEF | 2016 | DRR519079 |
| 2016_22 | OEF | 2016 | DRR519080 |
| 2016_23 | OEF | 2016 | DRR519081 |
| 2016_24 | OEF | 2016 | DRR519082 |
| 2016_25 | OEF | 2016 | DRR519083 |
| 2016_26 | OEF | 2016 | DRR519084 |
| 2016_27 | OEF | 2016 | DRR519085 |
| 2016_28 | OEF | 2016 | DRR519086 |
| 2016_29 | OEF | 2016 | DRR519087 |
| 2016_3 | OEF | 2016 | DRR519088 |
| 2016_30 | OEF | 2016 | DRR519089 |
| 2016_31 | OEF | 2016 | DRR519090 |
| 2016_32 | OEF | 2016 | DRR519091 |
| 2016_33 | OEF | 2016 | DRR519092 |
| 2016_34 | OEF | 2016 | DRR519093 |
| 2016_35 | OEF | 2016 | DRR519094 |
| 2016_36 | OEF | 2016 | DRR519095 |
| 2016_37 | OEF | 2016 | DRR519096 |
| 2016_38 | OEF | 2016 | DRR519097 |
| 2016_39 | OEF | 2016 | DRR519098 |
| 2016_40 | OEF | 2016 | DRR519099 |
| 2016_41 | OEF | 2016 | DRR519100 |
| 2016_42 | OEF | 2016 | DRR519101 |
| 2016_43 | OEF | 2016 | DRR519102 |
| 2016_44 | OEF | 2016 | DRR519103 |
| 2016_45 | OEF | 2016 | DRR519104 |
| 2016_46 | OEF | 2016 | DRR519105 |
| 2016_47 | OEF | 2016 | DRR519106 |
| 2016_48 | OEF | 2016 | DRR519107 |
| 2016_49 | OEF | 2016 | DRR519108 |
| 2016_5 | OEF | 2016 | DRR519109 |
| 2016_50 | OEF | 2016 | DRR519110 |
| 2016_51 | OEF | 2016 | DRR519111 |
| 2016_52 | OEF | 2016 | DRR519112 |
| 2016_53 | OEF | 2016 | DRR519113 |
| 2016_54 | OEF | 2016 | DRR519114 |
| 2016_55 | OEF | 2016 | DRR519115 |
| 2016_56 | OEF | 2016 | DRR519116 |
| 2016_57 | OEF | 2016 | DRR519117 |
| 2016_58 | OEF | 2016 | DRR519118 |
| 2016_59 | OEF | 2016 | DRR519119 |
| 2016_6 | OEF | 2016 | DRR519120 |
| 2016_60 | OEF | 2016 | DRR519121 |
| 2016_61 | OEF | 2016 | DRR519122 |
| 2016_62 | OEF | 2016 | DRR519123 |
| 2016_63 | OEF | 2016 | DRR519124 |
| 2016_64 | OEF | 2016 | DRR519125 |
| 2016_65 | OEF | 2016 | DRR519126 |
| 2016_66 | OEF | 2016 | DRR519127 |
| 2016_67 | OEF | 2016 | DRR519128 |
| 2016_68 | OEF | 2016 | DRR519129 |
| 2016_69 | OEF | 2016 | DRR519130 |
| 2016_7 | OEF | 2016 | DRR519131 |
| 2016_70 | OEF | 2016 | DRR519132 |
| 2016_71 | OEF | 2016 | DRR519133 |
| 2016_72 | OEF | 2016 | DRR519134 |
| 2016_73 | OEF | 2016 | DRR519135 |
| 2016_74 | OEF | 2016 | DRR519136 |
| 2016_75 | OEF | 2016 | DRR519137 |
| 2016_76 | OEF | 2016 | DRR519138 |
| 2016_77 | OEF | 2016 | DRR519139 |
| 2016_78 | OEF | 2016 | DRR519140 |
| 2016_79 | OEF | 2016 | DRR519141 |
| 2016_80 | OEF | 2016 | DRR519142 |
| 2016_81 | OEF | 2016 | DRR519143 |
| 2016_82 | OEF | 2016 | DRR519144 |
| 2016_83 | OEF | 2016 | DRR519145 |
| 2016_84 | OEF | 2016 | DRR519146 |
| 2016_85 | OEF | 2016 | DRR519147 |
| 2016_86 | OEF | 2016 | DRR519148 |
| 2016_87 | OEF | 2016 | DRR519149 |
| 2016_88 | OEF | 2016 | DRR519150 |
| 2016_89 | OEF | 2016 | DRR519151 |
| 2016_9 | OEF | 2016 | DRR519152 |
| 2016_90 | OEF | 2016 | DRR519153 |
| 2016_91 | OEF | 2016 | DRR519154 |
| 2016_92 | OEF | 2016 | DRR519155 |
| 2016_93 | OEF | 2016 | DRR519156 |
| 2016_94 | OEF | 2016 | DRR519157 |
| 2016_95 | OEF | 2016 | DRR519158 |
| 2016_96 | OEF | 2016 | DRR519159 |
| 2016_97 | OEF | 2016 | DRR519160 |
| 2016_98 | OEF | 2016 | DRR519161 |
| 2016_99 | OEF | 2016 | DRR519162 |
| 2017_1 | OEF | 2017 | DRR519163 |
| 2017_10 | OEF | 2017 | DRR519164 |
| 2017_11 | OEF | 2017 | DRR519165 |
| 2017_117 | OEF | 2017 | DRR519166 |
| 2017_12 | OEF | 2017 | DRR519167 |
| 2017_122 | OEF | 2017 | DRR519168 |
| 2017_123 | OEF | 2017 | DRR519169 |
| 2017_124 | OEF | 2017 | DRR519170 |
| 2017_128 | OEF | 2017 | DRR519171 |
| 2017_129 | OEF | 2017 | DRR519172 |
| 2017_13 | OEF | 2017 | DRR519173 |
| 2017_130 | OEF | 2017 | DRR519174 |
| 2017_14 | OEF | 2017 | DRR519175 |
| 2017_15 | OEF | 2017 | DRR519176 |
| 2017_16 | OEF | 2017 | DRR519177 |
| 2017_17 | OEF | 2017 | DRR519178 |
| 2017_18 | OEF | 2017 | DRR519179 |
| 2017_19 | OEF | 2017 | DRR519180 |
| 2017_2 | OEF | 2017 | DRR519181 |
| 2017_20 | OEF | 2017 | DRR519182 |
| 2017_21 | OEF | 2017 | DRR519183 |
| 2017_22 | OEF | 2017 | DRR519184 |
| 2017_23 | OEF | 2017 | DRR519185 |
| 2017_24 | OEF | 2017 | DRR519186 |
| 2017_25 | OEF | 2017 | DRR519187 |
| 2017_26 | OEF | 2017 | DRR519188 |
| 2017_27 | OEF | 2017 | DRR519189 |
| 2017_28 | OEF | 2017 | DRR519190 |
| 2017_29 | OEF | 2017 | DRR519191 |
| 2017_3 | OEF | 2017 | DRR519192 |
| 2017_30 | OEF | 2017 | DRR519193 |
| 2017_31 | OEF | 2017 | DRR519194 |
| 2017_32 | OEF | 2017 | DRR519195 |
| 2017_33 | OEF | 2017 | DRR519196 |
| 2017_34 | OEF | 2017 | DRR519197 |
| 2017_35 | OEF | 2017 | DRR519198 |
| 2017_36 | OEF | 2017 | DRR519199 |
| 2017_37 | OEF | 2017 | DRR519200 |
| 2017_38 | OEF | 2017 | DRR519201 |
| 2017_39 | OEF | 2017 | DRR519202 |
| 2017_4 | OEF | 2017 | DRR519203 |
| 2017_40 | OEF | 2017 | DRR519204 |
| 2017_41 | OEF | 2017 | DRR519205 |
| 2017_42 | OEF | 2017 | DRR519206 |
| 2017_43 | OEF | 2017 | DRR519207 |
| 2017_44 | OEF | 2017 | DRR519208 |
| 2017_45 | OEF | 2017 | DRR519209 |
| 2017_46 | OEF | 2017 | DRR519210 |
| 2017_47 | OEF | 2017 | DRR519211 |
| 2017_48 | OEF | 2017 | DRR519212 |
| 2017_49 | OEF | 2017 | DRR519213 |
| 2017_5 | OEF | 2017 | DRR519214 |
| 2017_50 | OEF | 2017 | DRR519215 |
| 2017_51 | OEF | 2017 | DRR519216 |
| 2017_52 | OEF | 2017 | DRR519217 |
| 2017_53 | OEF | 2017 | DRR519218 |
| 2017_54 | OEF | 2017 | DRR519219 |
| 2017_55 | OEF | 2017 | DRR519220 |
| 2017_56 | OEF | 2017 | DRR519221 |
| 2017_57 | OEF | 2017 | DRR519222 |
| 2017_58 | OEF | 2017 | DRR519223 |
| 2017_59 | OEF | 2017 | DRR519224 |
| 2017_6 | OEF | 2017 | DRR519225 |
| 2017_60 | OEF | 2017 | DRR519226 |
| 2017_61 | OEF | 2017 | DRR519227 |
| 2017_62 | OEF | 2017 | DRR519228 |
| 2017_63 | OEF | 2017 | DRR519229 |
| 2017_64 | OEF | 2017 | DRR519230 |
| 2017_65 | OEF | 2017 | DRR519231 |
| 2017_66 | OEF | 2017 | DRR519232 |
| 2017_67 | OEF | 2017 | DRR519233 |
| 2017_68 | OEF | 2017 | DRR519234 |
| 2017_69 | OEF | 2017 | DRR519235 |
| 2017_7 | OEF | 2017 | DRR519236 |
| 2017_70 | OEF | 2017 | DRR519237 |
| 2017_71 | OEF | 2017 | DRR519238 |
| 2017_72 | OEF | 2017 | DRR519239 |
| 2017_73 | OEF | 2017 | DRR519240 |
| 2017_74 | OEF | 2017 | DRR519241 |
| 2017_75 | OEF | 2017 | DRR519242 |
| 2017_76 | OEF | 2017 | DRR519243 |
| 2017_77 | OEF | 2017 | DRR519244 |
| 2017_78 | OEF | 2017 | DRR519245 |
| 2017_79 | OEF | 2017 | DRR519246 |
| 2017_8 | OEF | 2017 | DRR519247 |
| 2017_80 | OEF | 2017 | DRR519248 |
| 2017_81 | OEF | 2017 | DRR519249 |
| 2017_82 | OEF | 2017 | DRR519250 |
| 2017_83 | OEF | 2017 | DRR519251 |
| 2017_85 | OEF | 2017 | DRR519252 |
| 2017_86 | OEF | 2017 | DRR519253 |
| 2017_87 | OEF | 2017 | DRR519254 |
| 2017_88 | OEF | 2017 | DRR519255 |
| 2017_9 | OEF | 2017 | DRR519256 |
| 2017_91 | OEF | 2017 | DRR519257 |
| 2017_92 | OEF | 2017 | DRR519258 |
| 2017_93 | OEF | 2017 | DRR519259 |
| 2017_96 | OEF | 2017 | DRR519260 |
| 2017_97 | OEF | 2017 | DRR519261 |
| 2017_98 | OEF | 2017 | DRR519262 |
| FGP_21 | FGP | 2021 | DRR519263 |
| FGP_22 | FGP | 2021 | DRR519264 |
| FGP_23 | FGP | 2021 | DRR519265 |
| FGP_24 | FGP | 2021 | DRR519266 |
| FGP_25 | FGP | 2021 | DRR519267 |
| FGP_26 | FGP | 2021 | DRR519268 |
| FGP_27 | FGP | 2021 | DRR519269 |
| FGP_28 | FGP | 2021 | DRR519270 |
| FGP_29 | FGP | 2021 | DRR519271 |
| FGP_30 | FGP | 2021 | DRR519272 |
| FGP_31 | FGP | 2021 | DRR519273 |
| FGP_32 | FGP | 2021 | DRR519274 |
| FGP_33 | FGP | 2021 | DRR519275 |
| FGP_34 | FGP | 2021 | DRR519276 |
| FGP_35 | FGP | 2021 | DRR519277 |
| FGP_36 | FGP | 2021 | DRR519278 |
| FGP_37 | FGP | 2021 | DRR519279 |
| FGP_38 | FGP | 2021 | DRR519280 |
| FGP_39 | FGP | 2021 | DRR519281 |
| FGP_40 | FGP | 2021 | DRR519282 |
| FGP_41 | FGP | 2021 | DRR519283 |
| FGP_42 | FGP | 2021 | DRR519284 |
| FGP_43 | FGP | 2021 | DRR519285 |
| FGP_44 | FGP | 2021 | DRR519286 |
| FGP_45 | FGP | 2021 | DRR519287 |
| KGP_47 | KGP | 2021 | DRR519288 |
| KGP_48 | KGP | 2021 | DRR519289 |
| KGP_49 | KGP | 2021 | DRR519290 |
| KGP_50 | KGP | 2021 | DRR519291 |
| KGP_61 | KGP | 2021 | DRR519292 |
| KGP_62 | KGP | 2021 | DRR519293 |
| KGP_63 | KGP | 2021 | DRR519294 |
| KGP_64 | KGP | 2021 | DRR519295 |
| KGP_65 | KGP | 2021 | DRR519296 |
| KGP_66 | KGP | 2021 | DRR519297 |
| KGP_67 | KGP | 2021 | DRR519298 |
| KGP_68 | KGP | 2021 | DRR519299 |
| KGP_69 | KGP | 2021 | DRR519300 |
| KGP_70 | KGP | 2021 | DRR519301 |

|  | **Table S2.** Genetic distance (*F*_ST_) among Japanese emu populations. | | | | | |
| --- | --- | --- | --- | --- | --- | --- |
|  |  | TSP | OEF2016 | OEF2017 | FGP | KGP |
|  | TSP | - |  |  |  |  |
|  | OEF2016 | 0.009 | - |  |  |  |
|  | OEF2017 | 0.007 | 0.004 | - |  |  |
|  | FGP | 0.020 | 0.010 | 0.008 | - |  |
|  | KGP | 0.037 | 0.010 | 0.008 | 0.016 | - |
|  | TSP, OEF2016/2017, FGP, and KGP represents Tohoku Safari Park, Okhotsk emu fam, Fuji Kachoen Garden Park, and Kakegawa Kachoen Garden Park, respectively. | | | | | |
